# Changes in diet and supplement use in dogs with cancer

**DOI:** 10.1111/jvim.16825

**Published:** 2023-08-09

**Authors:** Matthew L. Kramer, Jennifer A. Larsen, Michael S. Kent

**Affiliations:** ^1^ Department of Surgical and Radiological Sciences, School of Veterinary Medicine University of California, Davis Davis California USA; ^2^ Department of Molecular Biosciences, School of Veterinary Medicine University of California, Davis Davis California USA

**Keywords:** dog, home‐prepared, informational resources, nutrition, oncology

## Abstract

**Background:**

Many dog owners alter their dog's nutritional regimen after a diagnosis of cancer. There are limited data as to specific changes made and reasons behind these changes.

**Hypothesis/Objectives:**

To collect updated and detailed data on changes made by owners to their dog's diet and supplements after a cancer diagnosis.

**Animals:**

Responses were collected from a survey of dog owners who brought their dogs to the UC Davis Veterinary Medical Teaching Hospital's Oncology Service for the first time after a cancer diagnosis. Dogs with recurrence or presenting for a second type of cancer were excluded.

**Methods:**

Eligible owners were surveyed between December 2020 and March 2022. The survey contained 62 questions regarding diet, supplement use, and treats, and how these were altered after a cancer diagnosis. Responses were matched to medical record data.

**Results:**

One hundred twenty‐eight surveys were retained for analysis, including 120 respondents that completed the survey. In response to a cancer diagnosis, 54.8% (95% CI; 45.7%‐63.8%) of owners altered diets or supplements or both. The most common informational resource for dog diets was veterinarians (53.9%). Usage of home‐prepared foods significantly increased after a cancer diagnosis (*P* = .03). There was no significant difference in commercial diet usage before or after a diagnosis (*P* = .25). Joint support products were the most common supplements given both before (37.4%) and after (35.0%) diagnosis.

**Conclusions and Clinical Importance:**

Many dog owners alter their dog's nutritional intake after a cancer diagnosis. These owners should be provided information relating to commonly observed alterations, including home‐prepared foods and supplements.

**Abbreviations**
BCS: body condition score
CBD: cannabidiol
DCM: dilated cardiomyopathy
Dx: diagnosis
THC: tetrahydrocannabinol

## 1 INTRODUCTION

Cancer is the leading cause of overall dog deaths with up to 27% of dog deaths attributed to this disease.^1^ This risk is highest for large breed dogs and those over 10 years of age.^2^, ^3^ Cancer and its treatments cause disruptions in nutritional status, such as loss of appetite and cachexia in many species.^4^, ^5^ In humans undergoing or surviving beyond treatment, evidence for the effectiveness of dietary strategies is inconsistent, which might reflect the complexity of the relationships among various nutritional factors, cancer biology, and both general and cancer‐specific outcomes. Overall, research data in humans suggest general healthy diet and lifestyle measures are beneficial after diagnosis and during treatment of cancer, including healthy weight management, exercise, increased fruit and vegetable consumption, and avoidance of red or processed meats.^6^, ^7^ Little evidence is available to support using specific nutritional strategies for dogs with cancer. Nonetheless, recommendations for specific commercial diets as well as home‐prepared diet recipes for dogs with cancer are readily accessible by pet owners, although the latter do not typically meet nutritional standards.^8^, ^9^, ^10^ Dogs with cancer are more likely to receive home‐prepared foods, with more than half of diet alterations after a cancer diagnosis involving the addition of home‐prepared components.^11^, ^12^ Owners of dogs with cancer are more likely to give supplements.^12^, ^13^

Given the importance of nutrition in dogs with cancer, and because owners might change the diets after diagnosis, it is important that clinicians collect information on what owners are feeding their dogs and how a cancer diagnosis affects this. There is limited data on alterations relating to supplement usage or specific diet strategies, such as grain‐free or organic. Diet history information, including supplement and treat use, is essential for performing the nutritional assessment, and understanding why certain changes are being made is important for client counseling and to develop sound treatment plans.^14^ This is especially relevant in cases where owners are switching to potentially unbalanced home‐prepared diets or adding raw animal products that carry the risk of contamination with pathogenic bacteria.^15^

This study sought to determine how dog owners altered diet and supplement usage in response to a cancer diagnosis. Owners were surveyed on diets and supplements given both before and after diagnosis, in addition to current treat usage. Reasons for alterations as well as informational resources used by owners of dogs with cancer were also queried. Based on previous studies, we expected that the most common changes would involve the initiation of supplements as well as the reduction or discontinuation of commercial diet products in favor of home‐prepared foods.

## 2 MATERIALS AND METHODS

This study included a cross‐sectional, internet‐based survey of dog owners with linkage to the dog's electronic medical record. The project proposal and survey outline were submitted to the Institutional Review Board at the University of California, Davis which determined that a formal review was not required (Institutional Review Board ID Number 1689098‐1).

### 2.1 Study cohort

Dog owners were surveyed if their dog presented, for the first time, to the UC Davis Veterinary Medical Teaching Hospital's Oncology Service for the treatment or further diagnosis of a tumor between December of 2020 and March of 2022. Dogs presenting to the service for the first time with recurrence or that had a previous, different type of cancer were excluded. Surveys were sent out in groups every 2 to 3 weeks. During this period, we reviewed the appointment schedule for newly presenting dogs and sent out surveys to owners upon identifying recruitment eligibility.

A link to the Qualtrics survey (*Qualtrics*, Qualtrics, Provo, Utah, USA, 2022; Supporting Information, Item 1) was distributed to eligible dog owners via the email address that they provided during registration of their dog at the hospital. Consent to participate was obtained through the first question of the survey. Participants were then asked if they would prefer to take the survey by telephone; if the participant selected this option, the online survey would end, and we attempted to collect responses via telephone call. Owners were excluded if they did not consent to the survey.

### 2.2 Survey

**FIGURE 1:**
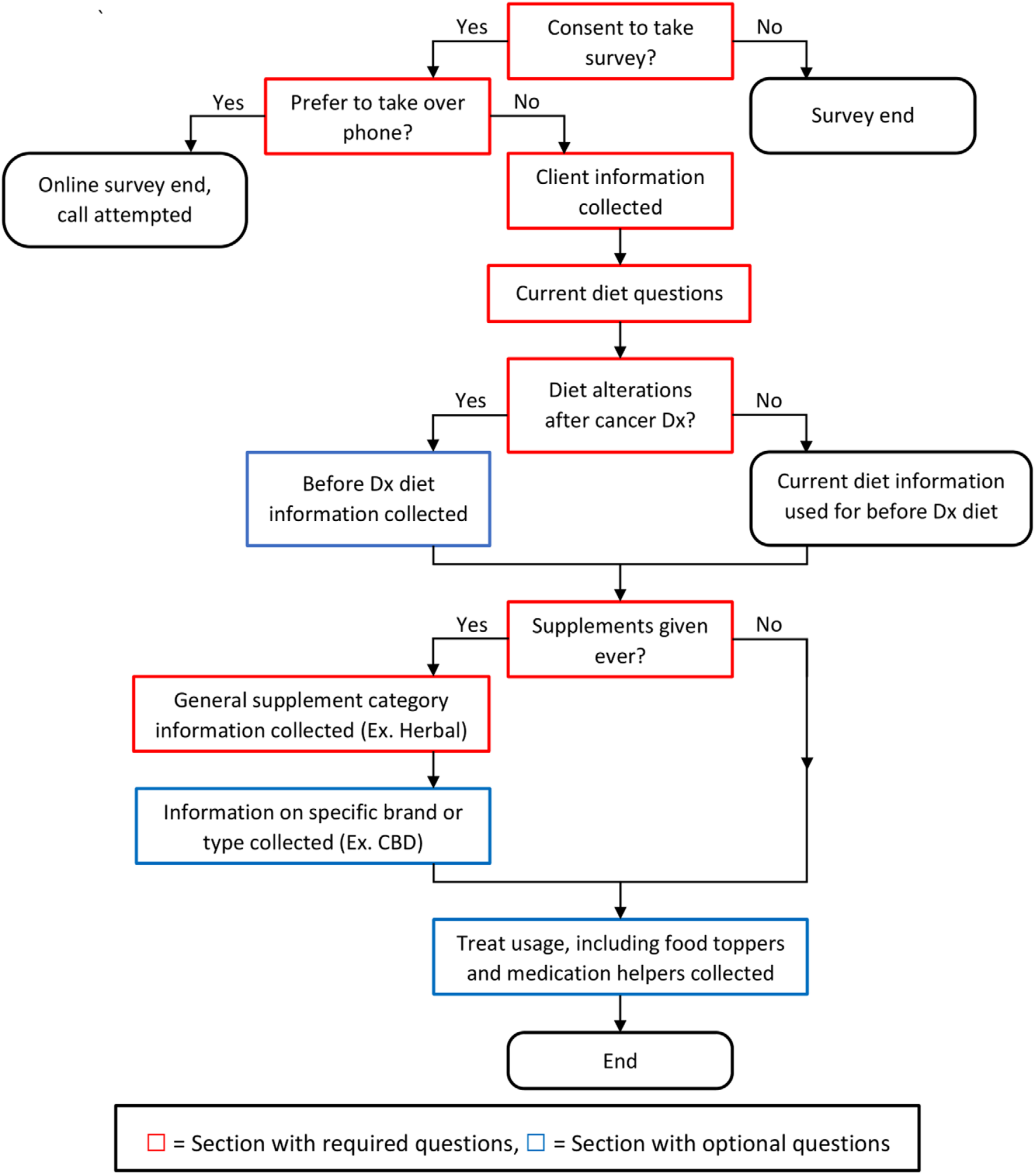
Overall survey flow. Sections in red required responses (respondent could not proceed without answering). Sections in blue had optional questions. CBD, cannabidiol; Dx, diagnosis.

An early draft of the survey was piloted with several dog owners. The final version of the survey had 62 possible questions, many of which were conditionally shown based on previous answers (Figure 1). Survey data were matched to each dog's medical record to collect information regarding signalment, diagnosis, time from diagnosis to survey response, and estimated household income based on census tract. The bulk of the survey consisted of 3 sections: diet, supplements, and treats. All eligible responses with information on diet, supplements, or treats were retained for analysis.

The diet section asked what owners were currently feeding their dogs, including specific characteristics (such as organic, raw, vegan, grain‐free, etc.) and if the diets were commercially available, home‐prepared, or a combination of the 2. For commercial diets, the brand name and form of diet (kibble, wet, etc.) was collected. For home‐prepared diets, a list of ingredients and the source of the recipe was requested. Participants also identified where they obtained information on choosing the diet.

For the supplement section, participants answered questions on which products were added or stopped in response to the cancer diagnosis, in addition to which supplements participants maintained both before and after the diagnosis. Participants were then asked where they obtained information on supplements and where they purchased these products for their dogs.

The treat section asked for the names of commonly given treats, how often the treat was given, and if the treat was used as a food topper or given with medications.

The diet and supplement sections collected both past and present information, whereas the treat section only collected present information.

To accommodate requests from initial survey testers and increase the overall survey experience, some questions did not force a response (Figure 1). This was to allow dog owners to complete the survey, even if they did not remember specifics for a particular question, and to reduce the total number of survey pages. Owners who altered their dog's diets because of a cancer diagnosis were given questions on the previous diet, but responses were not forced for brand (commercial diets), form of diet (commercial diets), ingredients (home‐prepared diets), or recipe source (home‐prepared diets). For the supplement section, responses were only forced for general supplement categories (eg, Vitamins, Minerals, etc.). Questions involving specific supplement types (such as specific herbs or brand names), informational resources used, and where supplements were bought did not force a response. The questions in the treat section did not force responses.

### 2.3 Medical record information

Data were collected from the medical record including date of birth, date of first visit to the oncology services, body weight at first visit (kg), sex, spay/neuter status, diagnosis, time from diagnosis to survey completion, breed, body condition score (BCS; based on a standard 9‐point scale^16^), appetite level at first visit (increased, normal, decreased), whether or not any signs of gastrointestinal disease were present at latest visit, and address. Address information was used to determine 2021 estimated census tract median family income.^17^

### 2.4 Statistical analysis

Survey data were exported into RStudio version 2022.12.0 (*RStudio Team*, RStudio, PBC, Boston, Massachusetts, USA, 2021) for statistical analysis using R version 4.2.2 (*R Core Team*, R Foundation of Statistical Computing, Vienna, Austria, 2021). Descriptive statistics were used to describe dog demographics. For diets and supplement use, reasons for changes and informational resources were reported as n values and percentages. Data were checked for normality using a Shapiro‐Wilk's test.

A logistic regression model was fitted to predict diet swapping based on information in the medical record or survey. We used the Akaike information criterion (AIC) to select from the possible variables of using veterinarians as a diet information resource, weight (kg), diagnosis group, estimated family income (determined by census tract), BCS, sex, and appetite at first visit (decreased/normal/increased). A *P* value of <.05 was considered statistically significant.

## 3 RESULTS

**FIGURE 2:**
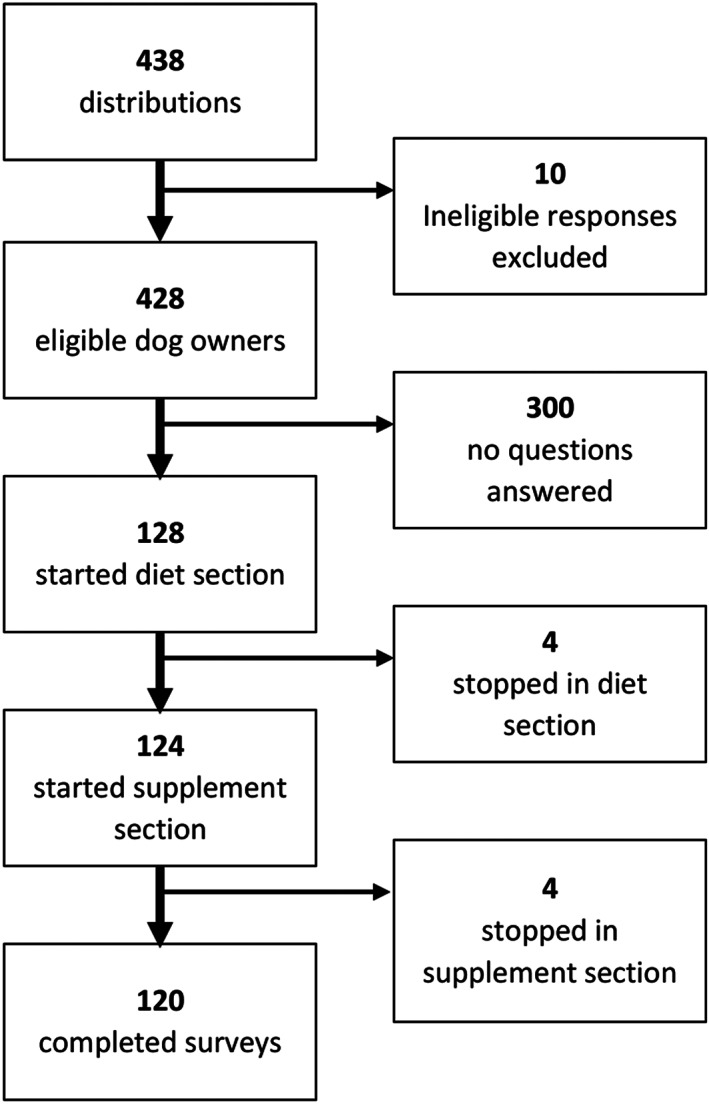
Flowchart of survey distributions and responses, including exclusions and where respondents stopped throughout the survey. A completed survey was any survey reaching and answering the final question, regardless of how many optional questions were answered.

The survey was distributed to 438 owners (Figure 2). Ten owners completed the survey but did not meet inclusion criteria, and these responses were omitted from analysis. One hundred twenty‐eight responses (29.2% response rate) included data for at least 1 nutrition‐related question and were retained for analysis. One hundred twenty of the responses were complete; any survey that reached the end was considered complete, regardless of optional questions answered. The median time from diagnosis to survey response was 61 days, with a range of 2 to 472 days (interquartile range: 25% = 42.5 days, 75% = 87 days).

Most questions forced responses and the reported sample sizes (n) will align with the number of respondents reaching a particular part of the survey (Figure 2). However, the sections that did not force a response had fluctuating sample sizes resulting from respondents proceeding through the survey without answering a particular question.

### 3.1 Overview

Out of the 128 dogs for which we had available survey data, 55.5% were male (3.9% intact vs 51.6% castrated) and 44.5% were female (3.9% intact vs 40.6% spayed). Omitting 2 dogs without date of birth in their medical records, the ages of 126 dogs were normally distributed (Shapiro‐Wilk's test, *P* = .4) with a mean of 9.9 years and a SD of 3.1 years. Omitting 1 dog without weight recorded at time of visit, 127 dogs had a median weight of 26.0 kg, with a range of 3.2 to 64.0 kg. All dogs had their breed recorded in the medical record, with 25.0% listed as mixed breed. Among purebred dogs (n = 96), the most common breeds were golden retrievers (9.4%), Labrador retrievers (8.3%), and German shepherds (7.3%). Only 102 (79.7%) of the 128 dogs had a BCS recorded in their medical record; the median BCS was a 5, with a minimum of 3 and a maximum of 9. Diagnoses for all 128 dogs were divided into 5 categories: epithelial origin tumors (27.3%), mesenchymal origin tumors (45.3%), round cell tumors (16.4%), benign tumors with clinical effect (2.3%) diagnosed as pituitary tumors or thymomas, and undefined masses (8.6%). For the 108 dogs with appetite described in the medical record at the time of visit, 13 had decreased appetite (12.0%), 91 had normal appetite (84.3%), and 4 had increased appetite (3.7%). 22.7% (n = 29) of all respondents (n = 128) reported any GI clinical signs in the time between initial presentation and survey completion.

Out of 128 respondents who answered diet information questions, 47.7% (95% CI; 38.8%‐56.7%) altered their dog's diet in response to a cancer diagnosis. Out of 124 respondents providing supplement information, 28.2% (95% CI; 20.5%‐37.0%) altered supplements in response to a cancer diagnosis, 20.9% (95% CI; 14.2%‐29.2%) altered both diets and supplements, and 54.8% (95% CI; 45.7%‐63.8%) altered diets and/or supplements.

### 3.2 Diet

The informational resource most widely used for both diets and supplements was veterinarians (Table 1). Among owners who changed their dog's diet (n = 61), the most common reason was loss of appetite (n = 22, 36%), followed by veterinarian recommendation (n = 17, 28%), and felt old diet was unhealthy (n = 14, 23%). Only 8% (n = 5) of respondents who changed their dog's diet did so for both reasons of loss of appetite and a veterinarian recommendation. Many owners selected the “other” option (n = 30, 49%). In this textbox, owners listed out reasons including previous dogs with cancer doing well on another diet, adding more meat, or reducing carbohydrates. Owners could select multiple options, with 28% (n = 17) of owners reporting multiple reasons.

**TABLE 1:** Information resources used by owners to select diets and supplements.

Information resource	Diets (n = 128)	Supplements (n = 81)
Veterinarian	0.54	0.68
Friend	0.20	0.17
Web (nonsocial media)	0.14	0.09
Pet store	0.12	0.09
Peer‐reviewed article	0.07	0.09
Social media	0.07	0.07
Breeder	0.04	0.02
Book	0.01	0.04
Other	0.25	0.17

*Note*: Respondents were required to answer with at least 1 resource but were able to indicate multiple. Values for diet are given as a proportion of all survey respondents (n = 128). Values for supplements are given as a proportion of respondents who reported giving supplements at any time (n = 81).

Before diagnosis (n = 128), 72.7% (n = 93) of dogs were fed commercial diets exclusively, 3.1% (n = 4) of dogs were fed home‐prepared diets exclusively, and 24.2% (n = 31) were fed a combination of commercial and home‐prepared diets. After diagnosis, 59.4% (n = 76) of dogs were fed commercial diets exclusively, 7.0% (n = 9) of dogs were fed home‐prepared diets exclusively, and 33.6% (n = 43) were fed a combination of commercial and home‐prepared diets. There was a significant increase in proportion of dogs fed at least some home‐prepared foods as part of their diet after the cancer diagnosis (27.3%‐40.6%, *P* = .03). However, there was no significant difference in the proportion of dogs fed any portion of their intake from a commercial diet after the cancer diagnosis (96.9% vs 93.0%, *P* = .25). However, many dogs fed commercial diets both before and after diagnosis changed commercial formulas; out of 49 respondents that provided this information for both timepoints, 23 switched diets (47%).

The most common recipe source for those making home‐prepared diets, both before and after a cancer diagnosis, was self‐formulation (Table 2). Of the 31 owners using home‐prepared diets before diagnosis, 45% (n = 14) reported using self‐formulation as the only recipe source. Of the 51 owners using home‐prepared diets after diagnosis, 39% (n = 20) only used self‐formulation. Veterinarians were the second most used resource both before and after a cancer diagnosis (Table 2).

**TABLE 2:** Recipe sources used for home‐prepared meals fed to dogs both before and after diagnosis of cancer, expressed as proportions.

Recipe sources	Before Dx (n = 31^a^)	After Dx (n = 51^a^)
Self‐formulated	0.58	0.51
Veterinarian	0.29	0.25
Book	0.06	0.10
Friend	0.06	0.08
Social media	0.06	0.04
Web (nonsocial media)	0.03	0.14
Pet store	0.03	0.02
Peer‐reviewed article	0.03	0
Other	0.19	0.24

*Note*: Respondents could indicate multiple sources used. Values are given as a proportion of respondents who utilized a specific source out of all respondents who gave home‐prepared meals for that time frame (eg, among 51 respondents feeding home‐prepared foods after diagnosis, 10% of them used books as a recipe source).

Abbreviation: Dx, diagnosis.

^a^ Owners feeding home‐prepared diets, but who did not provide a recipe source because of terminating the survey or leaving questions blank, were excluded (n = 4 before, n = 1 after).

The most common special diet category fed before diagnosis was grain‐free diets, and the most common after diagnosis was diets for a medical condition (Table 3). The term “natural” (including derivatives such as naturally) was included in the brand or product name of 6.9% (8/116) of commercial diets before diagnosis and 6.0% (7/117) of diets after, which was not significantly different (*P* = .99).

**TABLE 3:** Proportions of dogs receiving specialized diets, both before and after being diagnosed with cancer.

Special diets	Before Dx (n = 128)	After Dx (n = 128)
Grain‐free	0.22	0.14
Diet for medical condition	0.16	0.2
Raw	0.07	0.08
Organic	0.05	0.04
Vegan	0.02	0.02
Vegetarian	0.01	0.01
Other	0.21	0.26
None	0.45	0.47

*Note*: Respondents could indicate multiple special diet types. Values are given as the proportion of dogs fed special diets out of all surveyed respondents for a specified time frame (eg, 8% of all 128 dogs were fed raw food after diagnosis).

Abbreviation: Dx, diagnosis.

### 3.3 Supplement use

An overview of supplements given, including proportions given both before and after diagnosis, is presented in Table 4. Out of 124 respondents providing full or partial supplement information, 85 owners (68.5%) reported supplement use at any time, including 70 (56.5%) owners who reported supplement usage before diagnosis and 76 (61.3%) who reported supplement usage after diagnosis. There was no significant difference in number of dogs receiving supplements before or after a diagnosis (56.5% vs 61.3%, *P* = .52). The most common supplements were joint support products (37.4% before diagnosis and 35.0% after, total respondent n = 123). The most commonly added supplements after diagnosis were herbal products (14 of the 123 owners who reached this part of the survey). Out of the 14 owners who added herbal supplements, 12 owners provided specific types; among these owners, 7 reported adding mushroom supplements, and 4 reported adding cannabidiol (CBD) or tetrahydrocannabinol (THC). Among 83 respondents who specified where supplements were purchased, they were most likely to buy some or all of them online (61%), followed by veterinary offices (25%) and pet stores (18%). Other responses included warehouse stores (13%), pharmacies (6%), dispensaries (5%), or other locations (7%).

**TABLE 4:** Supplement usage by type, reported using proportions for both before and after a cancer diagnosis.

Category	Before Dx	After Dx
Any supplements (n = 124)	0.56	0.61
Vitamin (n = 123)	0.07	0.11
Mineral (n = 123)	0.03	0.05
Probiotic (n = 123)	0.11	0.14
Fatty acids (n = 123)	0.19	0.24
Herbal (n = 123)	0.07	0.16
Joint (n = 123)	0.37	0.35
Other (n = 123)	0.11	0.15

*Note*: Respondents could indicate multiple supplement types. One respondent terminated their survey before giving details on specific types. Values are given as the proportion of dogs given specific supplements types out of all survey respondents for a specified time frame.

Abbreviation: Dx, diagnosis.

### 3.4 Treats

Of 120 owners responding to the treat section of the survey, most reported giving treats (94.2%, n = 113). Among owners who gave treats, 23.9% (n = 26) used them as a food topper (total respondents n = 109, 11 owners who gave treats skipped this question without answering) and 47.7% (n = 53) gave treats alongside medications (total respondents n = 111, 9 owners who gave treats skipped this question without answering).

### 3.5 Modeling

Using the AIC as the criterion for our model to predict diet alterations, backwards‐forwards model selection yielded a logit model with weight (kg) and estimated family income (using the census tract median) as the 2 variables. Only estimated family income was significant (*P* = .03), and a new logit model was created using only this variable. Our model predicts that for every 10 000 dollars more of estimated family income, the chance of the owner swapping the diet is multiplied by 0.89.

## 4 DISCUSSION

This study examined owner decisions regarding diet and supplement alterations after a cancer diagnosis. The average age of dogs in this sample, around 10 years old, is similar to what has been reported in other studies for the age of development of cancer.^2^, ^18^ Given that older dogs have a higher incidence of degenerative joint disease, this might be related to the finding that joint support products were the most commonly used supplements, both before and after a cancer diagnosis; this is consistent with supplement use in dogs with cancer.^12^ For total number of dogs receiving supplements, our data showed no significant change after a cancer diagnosis. Dogs with cancer are more likely to receive supplements than healthy dogs in the general population,^11^ which was not evaluated in this study. Our findings might also reflect the owner's focus on supporting their dog during chemotherapy and other treatments during the period of time close to diagnosis.

Some owners added supplement products after diagnosis. Mushroom‐based and CBD/THC supplements were added at the highest rates after a cancer diagnosis; this is consistent with these supplements being more commonly given to dogs with cancer when compared to healthy dogs.^11^ This helps to confirm that some of these differences are attributable to a cancer diagnosis, rather than solely because of other factors such as age. Although mushrooms have anti‐cancer potential in mice,^19^, ^20^ this has not been shown in dogs.^21^ Similarly, although CBD use is common and has some efficacy in inducing apoptosis and perturbing mitochondrial function in canine glioma cell lines,^22^ there are currently no studies on its anti‐cancer effect in vivo. Furthermore, the addition of CBD to treats, foods, and supplements intended for animals is not allowed by the United States Food and Drug Administration and remains an illegal practice, which might have led to underreporting in this survey.^23^

Another potential instance of underreporting was loss of appetite; more owners reported loss of appetite in the survey than during their initial visit to the oncology service. However, this could also be the result of appetite normalizing before the visit, or appetite loss occurring between the visit and survey completion.

The present study showed an increase in the use of home‐prepared foods after a cancer diagnosis. We predicted this increase before conducting the survey, and this increase was in‐line with the results of a previous study.^12^ An increase in feeding of home‐prepared foods is similarly reported when comparing the general dog population to those with cancer.^11^ The increased use of these foods could potentially lead to inadequate nutrient intake, given that home‐prepared diets are commonly unbalanced.^8^, ^9^, ^10^ This concern is compounded by self‐formulation of recipes, which was the most common source for home‐prepared diets in the present study. However, this could be skewed by owners who added just a couple self‐selected ingredients to supplement a commercial diet, rather than fully developed recipes, since many owners gave home‐prepared meals in conjunction with commercial diets.

One strategy to address this potential problem would be referral to a board‐certified veterinary nutritionist to ensure any home‐prepared diet is complete and balanced. An alternative strategy could be to discuss with the owner their concerns with commercial pet foods. Collecting a comprehensive nutritional history is not only important for ensuring dietary needs are met, but the conversation could lead to discussion regarding perceived problems of commercial pet foods.

The current study did not find the accompanying decrease in commercial diets that has been shown elsewhere^12^ with the vast majority of owners using a commercial diet for part or all of their dog's foods. As our sample comprised dogs with a recent diagnosis of cancer, this might suggest that inclusion of home‐prepared elements precedes the complete exclusion of commercial diets, and our survey was conducted too close to the time of diagnosis to find exclusion of commercial diets. However, for owners feeding a commercial diet both before and after diagnosis, nearly half stopped feeding the pre‐cancer diagnosis diet. It is possible that our sample would ultimately have stayed on their second commercial diet, rather than eliminating commercial elements entirely.

Among owners feeding commercial diets, we found a decrease in the use of grain‐free foods, from 22% to 14% among all 128 respondents, after a cancer diagnosis. While this could seem contrary to the concerns of some owners regarding the role of carbohydrate in promoting cancer progression,^8^ possible benefits of the low carbohydrate approach have not been supported by any studies. Further, grain‐free diets can be lower, similar, or higher in carbohydrate content compared to other diet categories.^8^, ^24^ There has been considerable attention to the association between dilated cardiomyopathy (DCM) in dogs and the use of grain‐free diets,^25^, ^26^ and both veterinarians and pet owners might have increased awareness of this issue. Regardless, given that more than 1 in 5 dogs in the present study were fed a grain‐free diet before a cancer diagnosis, this data highlights the need for clinicians to discuss the risk of diet‐associated DCM with all dog owners.

The most common informational resource for diets and supplements was veterinarians, similar to previous studies for dogs.^11^, ^12^, ^27^ Veterinarians are a key resource for providing nutritional information, especially after a cancer diagnosis when veterinarians are actively involved with care, and around three quarters of pet owners believe a change is necessary.^12^ Additionally, as our data show, many dog owners do alter their dog's diet. These findings underscore the importance of collecting and assessing a thorough diet history. This enables effective client counseling by the veterinary care team to help guide and ensure the safe use of diets, treats, and supplement products. Our study did not differentiate whether veterinary advice was taken from general practitioners, cancer‐specialists, nutritionists, or elsewhere. Further specifying where owners receive information in a future study would be beneficial for understanding whose dietary advice pet owners value the most.

To assess which factors were most likely to result in diet changes, we created a logit model. Our logit model showed that 1 predictor of owners making diet changes was median census tract income, which lowers the chance of diet change as tract income increases. This suggests that people in wealthier areas might be less likely to alter their dog's diet in response to a diagnosis of cancer. Larger studies are warranted to confirm and further investigate this pattern.

One limitation of the current study was only involving dogs referred to a single hospital's oncology service. Coupled with time restrictions, this survey might not have recruited a large enough sample size to detect all of the patterns in nutritional alteration after a cancer diagnosis. Furthermore, dog owners within the geographical area of the survey might not be representative of the greater population of dogs and owners. Additionally, dog owners visiting oncology services are a subset of the overall dog owner population, meaning these data can only apply to dogs with a recent cancer diagnosis presenting for evaluation by a specialist. Any owners that decided not to pursue a second opinion or further treatment would not have visited the oncology service, and because of treatment associated costs, respondents to this survey could have more disposable income.

This study sought to capture a single snapshot in time, namely, when a dog initially presented to an oncology service. We do not know if this sample of dogs would have eventually shown similar or different patterns than other studies, such as exclusion of commercial diets and using social media groups for dietary and supplement recommendations. It is also possible that these owners would either revert to previously fed diets and supplements or make more extreme changes after treatment.

Although we attempted to capture the time‐point shortly after diagnosis, there was still a median delay of 61 days from diagnosis to survey. This is likely due the nature of online survey distributions, and the wait to get an oncology appointment which was exacerbated by the pandemic. Additionally, some dogs attempted cancer‐related treatments elsewhere before presenting to the oncology service. As a result, some dogs were already undergoing or finished treatments at the time of taking the survey, some of which might have caused gastrointestinal issues before survey completion. Nonetheless, we feel that the timeframe from diagnosis to survey enables us to capture additional nutritional changes beyond those simply because of an immediate medical need such as cancer and treatment related gastrointestinal signs. Further study is warranted into how specific treatments might result in changes to what owners feed their dogs.

This study also tried to balance the quality and completeness of data obtained with respondents' time and willingness to complete a lengthy survey. One concern was that adding too many questions would result in many owners not reaching the end of the survey. Since owners who made changes were asked additional questions, we felt these owners would disproportionately fail to reach the end of the survey, possibly skewing results.

Another consideration in interpreting the results of this study was if owners who changed their dog's diet or supplements could recall what was previously given. Based on initial piloting of the survey, some owners did not recall their dog's previous diets and supplements and were frustrated by the survey. As a result, the survey program did not force a response for these questions. This was done to ensure owners who did not remember previous nutritional information would be able to complete the survey without guessing unknowns. While we feel this goal was achieved, it is also likely that some owners who remembered simply skipped past these questions for the sake of time.

This study strived to be inclusive to all answers by providing textboxes, often referred to as “other” within the survey, if the owner felt the listed multiple‐choice options for a question did not apply. However, as the owners largely filled out the survey online by themselves, many either did not list what we were looking for, or possibly used the textbox as an additional place to put information, rather than intending to respond with “other.” These factors limited the value of the free text responses, and we feel that studies in the future could avoid these issues by either limiting free text responses in favor of more comprehensive multiple‐choice options or by administering the survey in person.

Overall, many dog owners make alterations to diet or supplements after their dog has been diagnosed with cancer. Clinicians should counsel owners regarding cancer treatment and its relation to nutrition to assess the current diet and enable educated decisions for any changes. Topics of focus could include discussing owner concerns regarding commercial diets, formulation of home‐prepared diets, and the use of certain herbal supplements, including mushrooms and CBD.

## CONFLICT OF INTEREST DECLARATION

Authors declare no conflict of interest.

## OFF‐LABEL ANTIMICROBIAL DECLARATION

Authors declare no off‐label use of antimicrobials.

## INSTITUTIONAL ANIMAL CARE AND USE COMMITTEE (IACUC) OR OTHER APPROVAL DECLARATION

Owners were given a brief description of information collected and project goals at the start of the survey, after which consent was obtained. No formal review was required.

## HUMAN ETHICS APPROVAL DECLARATION

The Institutional Review Board at the University of California, Davis determined that a formal review was not required (Institutional Review Board ID Number 1689098‐1).

## Supporting information


**Data S1:** Supplementary Information.
