# Supplementary material for: Changes in diet and supplement use in dogs with cancer
Source: J Vet Intern Med. 2023 Aug 9;37(5):1830–8. doi: 10.1111/jvim.16825 (PMC10473009; doi:10.1111/jvim.16825)
Supplement: Supplementary file 1 — Data S1: Supplementary Information. [file JVIM-37-1830-s001.pdf]

UC DAVIS
UNIVERSITY OF CALIFORNIA

Consent

Thank you for participating in this study. The following survey is regarding your dog who recently visited the UC Davis Veterinary Medical Teaching Hospital.

This survey will ask questions about your dog's diet, supplements, and treats (including foods used to give medications or as food toppers).

We will match your answers to your dog's information and diagnosis to analyze the results, but we will not put this information in the medical record or share this information with your dog's veterinarian.

This study is being conducted to find trends in diet and supplements. This data will not affect your dog's care. Principal Investigators: Dr. Michael Kent and Dr. Jennifer Larsen

Do you consent to participate in this survey?

Yes

No

Would you prefer to answer this survey by phone call?

Yes (Please enter your phone number and good times to call)

No (Continue to rest of survey)

Medical Information

The following information will be used to match your survey to your dog's medical information.

Your Name:

First

Last

Your Dog's Name:

Date of Last Visit to the UC Davis Veterinary Medical Teaching Hospital:

	Month	Day	Year
Please select:			

If you know your dog's medical record number, please write it below:

Diet - Basic

The following questions will be on your dog's current foods.

This does not include any medications, supplements, or treats your dog may be taking.

Has your dog's cancer diagnosis caused you to alter their diet? (*Multiple can be selected)*

Yes (Please give reason)

Loss of appetite

Felt old diet was unhealthy

Vet suggestion

Other (Please specify):

No

What resource(s) did you use to select your dog's foods? *Multiple can be selected.*

Vet Recommendation

Friend Suggestion

Newspaper

Book

Social Media (Please list website)

Blog (Please list blog name)

Online Forum (Please list forum name)

Website (Please list website name)

Breeder

Pet Store

Peer-reviewed article

Other

Is your dog being fed commercially available dog foods, home-prepared meals, or both?

Commercially available

Home-prepared

Both commercially available and home-prepared

Please specify any special diets your dog is on (other than home-prepared):

Grain-free

Organic

Raw

Special diet for a medical condition

Vegan

Vegetarian (non-Vegan)

Other (Please specify)

None of the above

Diet - Specific

The following questions will be on your dog's current foods.

This does not include any medications, supplements, or treats your dog may be taking.

Please list the full name of all commercial dog foods you give your dog:
(Examples: "Hill's Science Diet Adult 7+ Dog Food - Chicken & Brown Rice", "Purina Pro Plan Small Breed Salmon & Rice Formula")

What types of commercial dog foods do you give your dog? *Multiple can be selected.*

Dry (Such as kibble)

Wet (Such as canned)

Fresh

Frozen

Freeze dried

Dehydrated

Other (Please specify)

Where did you get the recipe(s) for your dog's home-prepared meals? *Multiple can be selected.*

Vet Recommendation

Friend Suggestion

Newspaper

Book

Social Media

Blog

Online Forum

Online Article

Breeder

Pet Store

Peer-reviewed article

Self-formulated

Other (Please specify)

Please list the ingredients (commonly) used in your dog's home-prepared meals:

Which medical condition is your dog's diet for? *Multiple can be selected.*

Allergies

Anorexia

Arthritis

Back pain

Bladder stones

Cancer

Critical illness

Degenerative (myxomatous) mitral valve disease

Diabetes

Dilated cardiomyopathy

Elbow dysplasia

Epilepsy

Gastric dilation

Hemolytic anemia

Hip dysplasia

Hyperthyroidism

Hypothyroidism

Inflammatory bowel disease

Megaesophagus

Obesity

Osteochondritis dissecans

Pancreatitis

Panosteitis

Pulmonary hypertension

Renal disease

Retinal dysplasia

Thrombocytopenia

Thrombocytosis

Urinary incontinence

von Willebrand disease

Weight loss

Other

Diet - Past - Basic

The following questions will be on your dog's previous diet, prior to a cancer diagnosis.

This does not include any medications, supplements, or treats your dog used to take.

Was your dog previously being fed commercially available dog foods, home-prepared meals, or both?

Commercially available

Home-prepared

Both commercially available and home-prepared

Please specify any special diets your dog previously was on (other than home-prepared):

Grain-free

Organic

Raw

Special diet for a medical condition

Vegan

Vegetarian (non-Vegan)

Other (Please specify)

None of the above

Diet - Past - Specific

The following questions will be on your dog's previous diet, prior to a cancer diagnosis.

This does not include any medications, supplements, or treats your dog used to take.

Please list the full name of all commercial dog foods you previously gave your dog (if you remember):
(Examples: "Hill's Science Diet Adult 7+ Dog Food - Chicken & Brown Rice", "Purina Pro Plan Small Breed Salmon & Rice Formula")

What types of commercial dog foods did you previously give your dog (if you remember)? *Multiple can be selected.*

Dry (Such as kibble)

Wet (Such as canned)

Fresh

Frozen

Freeze dried

Dehydrated

Other (Please specify)

Where did you get the recipe(s) for your dog's old home-prepared meals (if you remember)? *Multiple can be selected.*

Vet Recommendation

Friend Suggestion

Newspaper

Book

Social Media

Blog

Online Forum

Online Article

Breeder

Pet Store

Peer-reviewed article

Self-formulated

Other (Please specify)

Please list the ingredients (commonly) used in your dog's old home-prepared meals (if you remember):

Which medical condition was your dog's previous diet for? *Multiple can be selected.*

Allergies

Anorexia

Arthritis

Back pain

Bladder stones

Cancer

Critical illness

Degenerative (myxomatous) mitral valve disease

Diabetes

Dilated cardiomyopathy

Elbow dysplasia

Epilepsy

Gastric dilation

Hemolytic anemia

Hip dysplasia

Hyperthyroidism

Hypothyroidism

Inflammatory bowel disease

Megaesophagus

Obesity

Osteochondritis dissecans

Pancreatitis

Panosteitis

Pulmonary hypertension

Renal disease

Retinal dysplasia

Thrombocytopenia

Thrombocytosis

Urinary incontinence

von Willebrand disease

Weight loss

Other

Supplement Changes

The following questions will be on the supplements given to your dog.

Supplements would include: fatty acid supplements, vitamins, minerals, herbal medications, and joint protectants (but not treats, food toppers, and foods used to give medications)

Which of the following is true of your dog's supplement usage? *Multiple can be selected*.

Added supplements because of a cancer diagnosis

Stopped supplements because of a cancer diagnosis

Maintained some supplements (both before and after diagnosis)

My dog has never received supplements

(If you give any) Where do you obtain your dog's supplements? *Multiple can be selected.*

Veterinarian

Pet store

Warehouse store

Online retailer

Pharmacy

Dispensary

Other (Please specify)

(If you give any) What resource(s) made you choose your dog's supplements? *Multiple can be selected.*

Vet Recommendation

Friend Suggestion

Newspaper

Book

Social Media (Please list website)

Blog (Please list blog name)

Online Forum (Please list forum name)

Website (Please list website name)

Breeder

Pet Store

Peer-reviewed article

Other (Please specify)

Supplements - Information

The following questions are based on supplements you ${lm://Field/3}.

Which types of supplements did you ${lm://Field/2}? *Multiple can be selected.*

Vitamins (Such as multivitamins, vitamin A, niacin, etc.)

Minerals (Such as calcium, magnesium, selenium, etc.)

Probiotics (Such as Calming Care, FortiFlora, Proviable, etc.)

Fatty Acids (Such as fish oil, flax seed, linoleic acid, etc.)

Herbal medications (Such as Echinacea, CBD, mushroom-based, etc.)

Joint support medications/supplements (Also known as chondroprotectives)

Other (Please specify the product name)

The following questions are based on supplements you ${lm://Field/3}.

Vitamin(s):
Please enter the product name, if multiple, put a comma between each one.

Mineral(s): *Multiple can be selected.*

Boron

Calcium

Chloride

Chromium

Cobalt

Copper

Fluorine

Iodine
Iron
Magnesium
Manganese
Molybdenum
Phosphorous
Selenium
Sodium
Sulphur
Zinc
Other (Please specify)

Probiotics: *Multiple can be selected.*

Calming Care
FortiFlora
Proviable
Visbiome
Other (Please specify the product name)

Fatty Acid(s):
Please enter the product name, if multiple, put a comma between each one.

Herbal Medication(s):

Anamu
Artemisinin
Astragalus
CBD (With active ingredient THC)
CBD (Without active ingredient THC)
Chamomile
Corydalis

Echinacea

Elderberry

Flax seed

Garlic

Ginger

Ginkgo

Ginseng

Graviola (Also known as soursop)

Mushroom-based

Olive-based

Sea moss

St. John’s wort

Turmeric

Valerian

Other (Please specify)

Joint support medications/supplements (Chondroprotective(s)):
Please enter the product name, if multiple, put a comma between each one.

Treats - Number

The following questions will be on treats (including items given as a food topper or along with medicine) that you give your dog.

Please enter information on up to 4 commonly given treats.

Name of first treat:

Is this given as a food topper?

Yes

No

Is this given with medications?

Yes

No

Name of second treat (if more than one given):

Is this given as a food topper?

Yes

No

Is this given with medications?

Yes

No

Name of third treat (if more than two given):

Is this given as a food topper?

Yes

No

Is this given with medications?

Yes

No

Name of fourth treat (if more than three given):

Is this given as a food topper?

Yes

No

Is this given with medications?

Yes

No

Post-Survey Contact

May we contact you for additional information on your responses if necessary?
Personal contact information will not be stored after contact.

Yes (Please enter preferred method of contact)

No

Powered by Qualtrics
